# Antimicrobial Efficacy of Aqueous Extracts of Abrus precatorius and Achyranthes aspera Against Streptococcus mutans and Candida albicans: An In Vitro Study

**DOI:** 10.7759/cureus.112063

**Published:** 2026-07-04

**Authors:** Srujana Zakkula, Averneni Premalatha, Sankeerthana Inteti, Palamani Bhagyalakshmi, Pavan Kumar Manyam, Tharuni Thammareddy

**Affiliations:** 1 Department of Prosthodontics, Government Dental College and Hospital, Vijayawada, IND; 2 Department of Dental Design, Tata Consultancy Services, Noida, IND; 3 Department of Prosthodontics, Drs. Sudha and Nageswara Rao Siddhartha Institute of Dental Sciences, Gannavaram, IND; 4 Department of Prosthodontics, St. Joseph Dental College, Duggirala, IND; 5 Department of Prosthodontics, Navodaya Dental Clinic, Kothagudem, IND

**Keywords:** abrus precatorius, achyranthes aspera, antimicrobial agents, candida albicans, streptococcus mutans

## Abstract

Background

Dental caries and oral candidiasis are among the most common oral diseases and are primarily associated with *Streptococcus mutans *and *Candida albicans*. Growing concerns regarding antimicrobial resistance and the adverse effects of synthetic antimicrobial agents have increased interest in plant-derived alternatives. This study evaluated and compared the antimicrobial efficacies of aqueous extracts of *Abrus precatorius* and *Achyranthes aspera* against *Streptococcus mutans *and *Candida albicans*.

Methodology

An in vitro experimental study was conducted using aqueous extracts from the leaves of *Abrus precatorius* and *Achyranthes aspera*. The antimicrobial activity against *Streptococcus mutans* (ATCC 25175) and *Candida albicans* (ATCC 10231) was assessed using the agar well diffusion method. Chlorhexidine and nystatin served as positive controls for antibacterial and antifungal testing, respectively, and distilled water served as the negative control. Minimum inhibitory concentrations (MICs) were determined using the broth microdilution method. Data were analyzed using one-way analysis of variance followed by Tukey’s post-hoc test and independent-samples t-test.

Results

Significant differences in antimicrobial activity were observed among the groups (p < 0.001). Against *Streptococcus mutans*, *Abrus precatorius* demonstrated a larger mean zone of inhibition (18.42 ± 1.58 mm) than *Achyranthes aspera* (15.16 ± 1.47 mm), although chlorhexidine exhibited the greatest antibacterial activity (24.31 ± 1.32 mm). Similarly, against *Candida albicans*, *Abrus precatorius* produced a larger inhibition zone (16.87 ± 1.36 mm) than *Achyranthes aspera* (13.92 ± 1.41 mm), whereas nystatin showed the highest antifungal activity (22.74 ± 1.24 mm). *Abrus precatorius* also demonstrated significantly lower MIC values against both microorganisms, indicating a greater antimicrobial potency.

Conclusions

Both aqueous plant extracts exhibited significant antibacterial and antifungal activity. *Abrus precatorius* showed superior antimicrobial efficacy compared to *Achyranthes aspera*; however, both extracts were less effective than the standard antimicrobial agents. These findings suggest the potential application of these herbal extracts in the development of oral healthcare products.

## Introduction

Dental caries and oral candidiasis are among the most prevalent oral diseases worldwide [1]. *Streptococcus mutans* is a principal cariogenic bacterium owing to its ability to adhere to tooth surfaces, form biofilms, and produce acids that demineralize dental hard tissues [2]. *Candida albicans* is an opportunistic fungal pathogen frequently associated with oral candidiasis, particularly in immunocompromised individuals and denture wearers [3]. The increasing emergence of antimicrobial resistance and adverse effects associated with the long-term use of synthetic antimicrobial agents have stimulated interest in plant-derived therapeutic agents.

Ayurvedic medicinal plants have long been recognized for their antimicrobial, anti-inflammatory, and therapeutic properties [4]. *Abrus precatorius* and *Achyranthes aspera* are traditional medicinal plants that possess a wide range of bioactive phytochemicals, including alkaloids, flavonoids, tannins, saponins, and glycosides, which may contribute to antimicrobial activity [5]. Although several medicinal plants have been investigated for their antimicrobial properties, comparative studies evaluating the aqueous extracts of *Abrus precatorius* and *Achyranthes aspera* against clinically relevant oral pathogens remain limited. Most previous investigations have focused on individual plant species, different extraction methods, or non-oral microorganisms, making direct comparisons difficult [4,5]. Moreover, aqueous extraction is particularly relevant for the development of safe, economical, and clinically translatable herbal formulations intended for oral use. Therefore, a comparative evaluation of these two traditionally used medicinal plants against *Streptococcus mutans *and *Candida albicans* may provide valuable preliminary evidence for future research on plant-based oral antimicrobial agents.

Therefore, evaluation of their antimicrobial potential may provide a scientific basis for the development of novel herbal oral healthcare products. The present in vitro study aimed to compare the antimicrobial efficacy of aqueous extracts of *Abrus precatorius* and *Achyranthes aspera* against *Streptococcus mutans* (ATCC 25175) and *Candida albicans* (ATCC 10231). The objectives were to assess and compare the zones of inhibition produced by the extracts, determine their minimum inhibitory concentrations (MICs), and compare their efficacy with that of standard positive and negative controls.

## Materials and methods

Study design and setting

This in vitro experimental study was conducted in the Department of Prosthodontics, St. Joseph’s Dental College, Duggirala, Eluru, Andhra Pradesh, India, from March 2024 to July 2024. No human participants, human biological specimens, patient data, or animal subjects were included in this study. Therefore, approval from the Institutional Ethics Committee was not required. All experimental procedures were conducted in accordance with institutional laboratory biosafety guidelines and standard microbiological practices.

Preparation of plant extracts

Fresh leaves of *Abrus precatorius *and *Achyranthes aspera* were collected from authenticated botanical sources and thoroughly washed under running distilled water to remove dust and impurities. The leaves were shade-dried at room temperature for approximately two weeks until complete dehydration was achieved. The dried plant material was pulverized into a fine powder using a laboratory grinder and stored in sterile airtight containers until extraction.

To prepare the aqueous extracts, 100 g of powdered plant material from each species was mixed with 1,000 mL of distilled water and heated at 60°C for four hours with intermittent stirring. The resulting solution was filtered through a sterile muslin cloth and subsequently through Whatman No. 1 filter paper to remove particulate matter. The filtrate was concentrated using a rotary evaporator under reduced pressure and further dried in a hot-air oven maintained at 40°C until a semi-solid extract was obtained. The concentrated extracts were stored in sterile amber-colored containers at 4°C until further use. Before antimicrobial testing, stock solutions of the extracts were prepared using sterile distilled water.

Study groups

The study consisted of four groups. Group I comprised the aqueous extract of *Abrus precatorius*; Group II comprised the aqueous extract of *Achyranthes aspera*; Group III served as the positive control and included 0.2% chlorhexidine gluconate for antibacterial testing against* Streptococcus mutans* and nystatin (100,000 IU/mL) for antifungal testing against *Candida albicans*; and Group IV consisted of sterile distilled water and served as the negative control.

Preparation of microbial cultures

Standard reference strains of *Streptococcus mutans* (ATCC 25175) and *Candida albicans* (ATCC 10231) were obtained from a certified microbial culture repository. *Streptococcus mutans* was cultured on brain-heart infusion agar, whereas* Candida albicans* was cultured on Sabouraud dextrose agar. Sterile cork borers were used to prepare wells measuring 6 mm in diameter in each agar plate. The aqueous plant extracts were prepared at a concentration of 100 mg/mL, and 100 µL of each extract was dispensed into the respective well using a sterile micropipette. The positive controls (0.2% chlorhexidine gluconate for *Streptococcus mutans* and nystatin for *Candida albicans*) and the negative control (sterile distilled water) were also dispensed in the same volume (100 µL). The plates were allowed to stand at room temperature for 30 minutes to facilitate diffusion of the test substances into the agar medium before incubation. The cultures were incubated under appropriate growth conditions to obtain fresh colonies. Microbial suspensions were prepared by transferring isolated colonies into sterile saline solution and adjusting the turbidity to match the 0.5 McFarland standard of approximately 1.5 × 10 colony-forming units/mL. The standardized suspensions were immediately used for antimicrobial testing.

Evaluation of antimicrobial activity using the agar well diffusion method

The antimicrobial activities of the plant extracts were evaluated using the agar well diffusion technique. Sterile Mueller-Hinton agar plates supplemented with 5% sheep blood were used for *Streptococcus mutans*, whereas Sabouraud dextrose agar plates were used for *Candida albicans*. Standardized microbial suspensions were evenly spread across the agar surface using sterile cotton swabs to achieve uniform inoculation.

Sterile cork borers were used to prepare wells measuring 6 mm in diameter on each agar plate. A predetermined volume of test solution was carefully dispensed into each well. The plates were allowed to stand at room temperature for 30 minutes to facilitate diffusion of the test substances into the agar medium. Subsequently, the plates inoculated with *Streptococcus mutans* were incubated at 37°C under microaerophilic conditions for 24 hours, while those inoculated with *Candida albicans* were incubated aerobically at 37°C for 24-48 hours. Following incubation, the diameter of the clear inhibition zone surrounding each well was measured in millimeters. Measurements were obtained using ImageJ software version 1.54 (National Institutes of Health, Bethesda, Maryland, USA), and the mean values of three independent measurements were recorded for analysis.

Determination of minimum inhibitory concentration

The MIC of each plant extract against both microorganisms was determined using the broth microdilution method. Serial two-fold dilutions of each extract were prepared in a sterile nutrient broth to obtain a range of concentrations. Equal volumes of standardized microbial suspensions were added to each dilution tube. Positive growth and sterile broth controls were included in each experimental run.

The inoculated tubes were incubated under appropriate conditions for each microorganism. After incubation, microbial growth was visually assessed based on turbidity. The lowest concentration of extract that completely inhibited visible growth was recorded as the MIC. All MIC determinations were performed in triplicate, and the average value was used for statistical analysis.

Sample size estimation

Sample size estimation was performed using G*Power software version 3.1.9.7 (Heinrich Heine University, Düsseldorf, Germany). Based on a previous study that evaluated the antimicrobial efficacy of herbal extracts against oral microorganisms, an effect size of 0.50, a significance level of 5%, and a statistical power of 80% were considered [6]. The minimum required sample size was calculated as 36 observations, corresponding to nine observations per study group. To compensate for potential experimental variability and improve reliability, 10 observations were included in each group.

Statistical analysis

Data were entered into Microsoft Excel 2021 (Microsoft Corp.n, Redmond, WA, USA) and analyzed using SPSS Statistics for Windows version 25.0 (IBM Corp., Armonk, NY, USA). Descriptive statistics are expressed as mean ± standard deviation for continuous variables. The normality of the data distribution was assessed using the Shapiro-Wilk test. Intergroup comparisons of the mean zones of inhibition were performed using one-way analysis of variance (ANOVA), followed by Tukey’s post-hoc test for multiple pairwise comparisons. Comparisons of MIC values between the two herbal extracts were performed using the independent-samples t-test. Differences were considered statistically significant at a p-value <0.05.

## Results

The antimicrobial efficacy of the aqueous extracts of *Abrus precatorius *and *Achyranthes aspera* was evaluated against *Streptococcus mutans* (ATCC 25175) and *Candida albicans (*ATCC 10231) using agar well diffusion and MIC assays. As shown in Table 1, significant differences were observed in the mean zone of inhibition of *Streptococcus mutans* among the study groups (F = 98.74, p < 0.001). The positive control, chlorhexidine, demonstrated the highest antibacterial activity with a mean inhibition zone of 24.31 ± 1.32 mm. Among the herbal extracts, *Abrus precatorius* exhibited a significantly larger inhibition zone (18.42 ± 1.58 mm) than *Achyranthes aspera* (15.16 ± 1.47 mm). The negative control group (distilled water) showed no inhibitory activity. These findings indicate that both herbal extracts possess antibacterial activity against *Streptococcus mutans*, with *Abrus precatorius* demonstrating superior efficacy.

**Table 1:** Mean zone of inhibition against Streptococcus mutans among the study groups. Data are presented as mean ± standard deviation (SD); intergroup comparison was performed using one-way analysis of variance (ANOVA); p < 0.05 was considered statistically significant; *: p < 0.001 indicates statistical significance.

Group	n	Mean ± SD (mm)	ANOVA F-value	P-value
Abrus precatorius extract	10	18.42 ± 1.58	98.74	<0.001*
Achyranthes aspera extract	10	15.16 ± 1.47		
Chlorhexidine (positive control)	10	24.31 ± 1.32		
Distilled water (negative control)	10	0.00 ± 0.00		

Table 2 presents the antifungal activities of the tested agents against *Candida albicans*. A statistically significant difference was observed between groups (F = 112.68, p < 0.001). Nystatin, used as the positive control, produced the largest mean zone of inhibition (22.74 ± 1.24 mm). Among the plant extracts, *Abrus precatorius* demonstrated greater antifungal activity with a mean inhibition zone of 16.87 ± 1.36 mm compared with 13.92 ± 1.41 mm for *Achyranthes aspera*. No inhibition was observed in the distilled water-treated group. Although both extracts exhibited antifungal properties, their efficacies were lower than that of nystatin.

**Table 2:** Mean zone of inhibition against Candida albicans among the study groups. Data are presented as mean ± standard deviation (SD); intergroup comparison was performed using one-way analysis of variance (ANOVA); p < 0.05 was considered statistically significant; *: p < 0.001 indicates statistical significance.

Group	n	Mean ± SD (mm)	ANOVA F-value	P-value
Abrus precatorius extract	10	16.87 ± 1.36	112.68	<0.001*
Achyranthes aspera extract	10	13.92 ± 1.41		
Nystatin (positive control)	10	22.74 ± 1.24		
Distilled water (negative control)	10	0.00 ± 0.00		

The MICs of the two herbal extracts against the tested microorganisms are summarized in Table 3. Against *Streptococcus mutans*, *Abrus precatorius* showed a significantly lower MIC value (1.78 ± 0.42 mg/mL) compared with *Achyranthes aspera* (3.52 ± 0.71 mg/mL) (p < 0.001). Similarly, against *Candida albicans*, *Abrus precatorius* demonstrated a lower MIC value (3.14 ± 0.68 mg/mL) than *Achyranthes aspera* (6.26 ± 1.08 mg/mL) (p < 0.001). The lower MIC values observed for *Abrus precatorius* indicated that smaller concentrations of the extract were sufficient to inhibit microbial growth, reflecting greater antimicrobial potency.

**Table 3:** Minimum inhibitory concentration values of the herbal extracts against Streptococcus mutans and Candida albicans. Data are presented as mean ± standard deviation (SD) and range; comparison between extracts was performed using the independent-samples t-test; p < 0.05 was considered statistically significant; *: p < 0.001 indicates statistical significance. MIC = minimum inhibitory concentration

Microorganism	Group	MIC range (mg/mL)	Mean MIC ± SD (mg/mL)	Test value	P-value
Streptococcus mutans	Abrus precatorius	1.25–2.50	1.78 ± 0.42	6.89	<0.001*
	Achyranthes aspera	2.50–5.00	3.52 ± 0.71		
Candida albicans	Abrus precatorius	2.50–5.00	3.14 ± 0.68	7.84	<0.001*
	Achyranthes aspera	5.00–10.00	6.26 ± 1.08		

Pairwise comparisons of antibacterial activity against *Streptococcus mutans* using Tukey’s post-hoc test are presented in Table 4. *Abrus precatorius* exhibited significantly greater antibacterial efficacy than *Achyranthes aspera* (mean difference = 3.26 mm, p < 0.001). Chlorhexidine produced significantly larger inhibition zones than both *Abrus precatorius* (mean difference = 5.89 mm, p < 0.001) and *Achyranthes aspera* (mean difference = 9.15 mm, p < 0.001). Furthermore, all the active treatment groups demonstrated significantly greater antimicrobial activity than the negative control group (p < 0.001).

**Table 4:** Pairwise comparison of antibacterial activity against Streptococcus mutans using Tukey’s post-hoc test. Pairwise comparisons were performed following one-way analysis of variance (ANOVA) using Tukey’s post-hoc test; data are presented as mean difference with 95% confidence interval (CI); p < 0.05 was considered statistically significant; *: p < 0.001 indicates statistical significance.

Comparison	Mean difference (mm)	95% CI	t value	P-value
Abrus precatorius vs. Achyranthes aspera	3.26	1.71 to 4.81	6.54	<0.001*
Abrus precatorius vs. chlorhexidine	-5.89	-7.44 to -4.34	11.82	<0.001*
Achyranthes aspera vs. Chlorhexidine	-9.15	-10.70 to -7.60	18.36	<0.001*
Abrus precatorius vs. distilled water	18.42	16.87 to 19.97	36.95	<0.001*
Achyranthes aspera vs. distilled water	15.16	13.61 to 16.71	30.41	<0.001*
Chlorhexidine vs. distilled water	24.31	22.76 to 25.86	48.77	<0.001*

The results of the pairwise comparisons of antifungal activity against *Candida albicans* are shown in Table 5. *Abrus precatorius* demonstrated significantly greater antifungal activity than *Achyranthes aspera* (mean difference = 2.95 mm, p < 0.001). Nystatin showed significantly larger inhibition zones than both *Abrus precatorius* (mean difference = 5.87 mm, p < 0.001) and *Achyranthes aspera* (mean difference = 8.82 mm, p < 0.001). Similar to the antibacterial findings, all active treatment groups exhibited significantly greater antifungal efficacy than the distilled water group (p < 0.001). Overall, both herbal extracts demonstrated significant antimicrobial activities against *Streptococcus mutans* and *Candida albicans*. However, *Abrus precatorius* consistently exhibited larger zones of inhibition and lower MIC values than *Achyranthes aspera*, indicating superior antibacterial and antifungal efficacy. Nevertheless, the standard antimicrobial agents chlorhexidine and nystatin remained significantly more effective than either herbal extract.

**Table 5:** Pairwise comparison of antifungal activity against Candida albicans using Tukey’s post-hoc test. Pairwise comparisons were performed following one-way analysis of variance (ANOVA) using Tukey’s post-hoc test; data are presented as mean difference with 95% confidence interval (CI); p < 0.05 was considered statistically significant; *: p < 0.001 indicates statistical significance.

Comparison	Mean difference (mm)	95% CI	t value	P-value
Abrus precatorius vs. Achyranthes aspera	2.95	1.46 to 4.44	6.03	<0.001*
Abrus precatorius vs. nystatin	-5.87	-7.36 to -4.38	12.01	<0.001*
Achyranthes aspera vs. nystatin	-8.82	-10.31 to -7.33	18.04	<0.001*
Abrus precatorius vs. distilled water	16.87	15.38 to 18.36	34.53	<0.001*
Achyranthes aspera vs. distilled water	13.92	12.43 to 15.41	28.50	<0.001*
Nystatin vs. distilled water	22.74	21.25 to 24.23	46.54	<0.001*

## Discussion

This in vitro study evaluated and compared the antimicrobial efficacy of aqueous extracts of *Abrus precatorius *and *Achyranthes aspera *against *Streptococcus mutans* and *Candida albicans*. The findings demonstrated that both herbal extracts exhibited significant antibacterial and antifungal activities; however, *Abrus precatorius* consistently produced larger zones of inhibition and lower MIC values than* Achyranthes aspera*. Nevertheless, the antimicrobial efficacy of both extracts remained lower than that of the standard agents, chlorhexidine and nystatin.

The observed inhibitory activity against *Streptococcus mutans *may be attributed to the presence of several bioactive phytoconstituents in both plants. *Abrus precatorius *contains alkaloids, flavonoids, triterpenoids, tannins, saponins, and glycosides that have been reported to interfere with bacterial cell wall synthesis, membrane permeability, and metabolic activity. Flavonoids and tannins can form complexes with extracellular proteins and bacterial cell walls, resulting in the disruption of microbial growth and biofilm formation [7-9]. As *Streptococcus mutans* plays a critical role in dental plaque formation and acid production, inhibition of its growth may contribute to the prevention of dental caries.

The superior antibacterial activity demonstrated by *Abrus precatorius *compared with *Achyranthes aspera* in the present study may be related to differences in the concentration and composition of phytochemicals present in the extracts [10]. Previous phytochemical analyses have shown that *Abrus precatorius* possesses high levels of phenolic compounds and flavonoids, which are known to exhibit potent antimicrobial effects [7-9,11]. The lower MIC values observed for *Abrus precatorius* further support its greater antimicrobial potency, as a lower concentration was sufficient to inhibit bacterial growth.

The antifungal activity observed against *Candida albicans *was also consistent with the phytochemical profiles of both plants. Saponins and phenolic compounds can disrupt fungal cell membrane integrity by interacting with membrane sterols, resulting in the leakage of intracellular contents and inhibition of fungal proliferation. The greater antifungal efficacy of *Abrus precatorius* may again be related to its rich phytochemical composition and synergistic interactions among its bioactive constituents [11]. Although nystatin exhibited significantly larger inhibition zones than both herbal extracts, the results suggested that these plants possess appreciable antifungal properties and may serve as potential adjunctive agents for oral fungal infections [12].

The findings of the present study are in agreement with those reported by Balaraman et al. [6], who demonstrated the significant antibacterial and antifungal activities of various Ayurvedic medicinal plants against oral pathogens. Similarly, Garaniya and Bapodra [7] reported that *Abrus precatorius *possesses considerable antimicrobial potential, owing to its abundance of bioactive phytochemicals. Previous studies have also documented the inhibitory effects of *Achyranthes aspera* extracts against both gram-positive bacteria and fungal species, supporting the findings of the present study [10,13].

The greater antimicrobial efficacy of *Abrus precatorius *observed in this study is also consistent with reports by Kumar et al. [14], who found that extracts of* Abrus precatorius* produced larger inhibition zones against oral and non-oral microbial species compared with several other medicinal plants. Similar observations have been reported by Kuo et al. [15], who attributed the antimicrobial activity of *Abrus precatorius* to the synergistic action of flavonoids, alkaloids, and tannins.

The antimicrobial activity of *Achyranthes aspera* observed in the present study is consistent with previous investigations that demonstrated its effectiveness against oral pathogens. Yadav et al. [5] reported that aqueous extracts of *Achyranthes aspera* exhibited significant antibacterial activity against *Streptococcus mutans,* supporting its potential role in the prevention of dental caries. Similarly, Jebashree et al. [16] evaluated various solvent extracts of medicinal plants against cariogenic microorganisms and found that ethyl acetate, ethanol, and methanol extracts of *Achyranthes aspera* exhibited inhibitory activity against both *Streptococcus mutans* and *Candida albicans*. The comparatively higher antimicrobial activity reported with organic solvent extracts suggests that the extraction methodology may influence the recovery of bioactive phytochemicals and, consequently, the antimicrobial efficacy of the plant. These findings support the results of the present study and further substantiate the therapeutic potential of *Achyranthes aspera* against oral bacterial and fungal pathogens.

From a clinical perspective, the growing concern regarding antimicrobial resistance and adverse effects associated with the long-term use of synthetic antimicrobial agents has increased the interest in herbal alternatives. The demonstrated efficacy of *Abrus precatorius* and *Achyranthes aspera* suggests their potential application in the development of herbal mouth rinses, dentifrices, gels, and other oral healthcare products to control cariogenic and opportunistic fungal microorganisms. Their natural origin, availability, and traditional medicinal uses further support their potential utility in preventive dentistry.

The present study has certain limitations. As an in vitro investigation, the findings may not fully replicate the complex oral environment, where factors such as saliva, multispecies biofilm architecture, host immune responses, and dynamic microbial interactions influence antimicrobial efficacy. Only aqueous extracts were evaluated; therefore, other extraction methods may yield different phytochemical profiles and antimicrobial activities. Furthermore, the study assessed only two oral microorganisms and did not investigate cytotoxicity on oral tissues, biofilm inhibition or eradication, phytochemical characterization, or the long-term antimicrobial effects of the extracts. Although aqueous leaf extracts of *Abrus precatorius* were evaluated in the present study, the safety of these extracts cannot be assumed based solely on their traditional medicinal use. Comprehensive toxicological and allergenicity assessments are essential before considering oral healthcare applications, particularly formulations such as mouthwashes, where accidental ingestion may occur. These evaluations are essential before considering clinical applications such as herbal mouthwashes. Future studies should investigate different extraction techniques, assess biocompatibility using appropriate oral cell lines, evaluate antibiofilm activity using established biofilm models, characterize the active phytochemical constituents, and conduct well-designed in vivo and clinical studies to validate the safety, efficacy, and therapeutic potential of these herbal extracts.

## Conclusions

Within the limitations of this in vitro study, the aqueous extracts of *Abrus precatorius *and *Achyranthes aspera* demonstrated preliminary antimicrobial activity against *Streptococcus mutans* and *Candida albicans*, with *Abrus precatorius* exhibiting greater antimicrobial efficacy than *Achyranthes aspera*. Although these findings suggest that the extracts possess promising antimicrobial potential, they should be considered preliminary and do not justify clinical application at this stage. Further studies involving phytochemical characterization, cytotoxicity assessment, antibiofilm evaluation, and well-designed in vivo and clinical investigations are necessary to establish their safety, efficacy, and therapeutic potential for oral healthcare applications.
